# Group B Strep: Successful Model of "From Science to Action"^1^

**DOI:** 10.3201/eid1011.040623_03

**Published:** 2004-11

**Authors:** Janine Cory, Stephanie Schrag, Mara J. Dinsmoor, Anne Schuchat

**Affiliations:** * Centers for Disease Control and Prevention, Atlanta, Georgia, USA;; † Evanston Northwestern Hospital, Evanston, Illinois, USA

**Keywords:** group B streptococcal disease, GBS, perinatal disease

This panel session on the prevention of perinatal group B streptococcal disease (GBS) offered three perspectives on the history of the disease as an emerging concern in the United States. The panel addressed how recent epidemiologic research has effected a change in screening policy and a reduction in disease incidence, as well as the ongoing and future challenges presented by this disease.

## Perspective on U.S. Transformation and Global GBS Needs

The session opened with an overview of GBS epidemiology and policy, demonstrating progress that has been made since screening recommendations were created in the early 1990s, including a consensus statement issued in 1996. Since then, GBS has dramatically declined. Further evidence led the Centers for Disease Control and Prevention to issue universal screening policy guidelines in 2002. The guidelines were a transition from the risk-based and screening-based strategies used earlier. As guidelines become more firmly entrenched as part of current obstetric care, new data must be collected to determine whether the trend of the past few years toward a plateau in screening efficacy, with particular distinctions between black and white women, will be eliminated by the new universal screening guidelines.

## Obstetric Perspective on GBS Progress and Remaining Challenges

An obstetrician's point of view on the history of GBS detection and treatment and the current challenges in enacting a change in policy was presented. While screening and treatment rates rise, do adverse effects of intrapartum antibiotics, including antibiotic resistance or neonatal *Escherichia coli* sepsis, also increase? Also reiterated was the need to continue to educate clinicians, both obstetricians and pediatricians, to help keep GBS identification and treatment in perspective.

## How Science, Advocacy, and Policy Help Women and Children

To offer a historic perspective of the successes and challenges posed by incorporating epidemiologic evidence into everyday policy, the session closed with a discussion of how political timing, advocacy groups, dedicated researchers, and skillful partnership development all have interacted simultaneously. As key surveillance revealed the epidemiologic picture of GBS, other factors played equally important roles in helping bring the science of GBS into the political limelight, which resulted in an active change in screening policy. GBS policy has the advantage of being firmly linked to a true reduction in disease; since 1993, >39,000 cases of GBS are estimated to have been prevented. As a working model of prevention, GBS policy continues to evolve to address ongoing issues.

